# General practice training and virtual communities of practice - a review of the literature

**DOI:** 10.1186/1471-2296-13-87

**Published:** 2012-08-21

**Authors:** Stephen Barnett, Sandra C Jones, Sue Bennett, Don Iverson, Andrew Bonney

**Affiliations:** 1 Graduate School of Medicine, University of Wollongong, Wollongong, NSW, 2522, Australia; 2 Centre for Health Initiatives, University of Wollongong, Wollongong, NSW, 2533, Australia; 3 Faculty of Education, University of Wollongong, Wollongong, NSW, 2522, Australia; 4 Health and Behavioural Sciences, University of Wollongong, Wollongong, NSW, 2522, Australia

**Keywords:** General practice, Training, Communities of practice

## Abstract

**Background:**

Good General Practice is essential for an effective health system. Good General Practice training is essential to sustain the workforce, however training for General Practice can be hampered by a number of pressures, including professional, structural and social isolation. General Practice trainees may be under more pressure than fully registered General Practitioners, and yet isolation can lead doctors to reduce hours and move away from rural practice. Virtual communities of practice (VCoPs) in business have been shown to be effective in improving knowledge sharing, thus reducing professional and structural isolation. This literature review will critically examine the current evidence relevant to virtual communities of practice in General Practice training, identify evidence-based principles that might guide their construction and suggest further avenues for research.

**Methods:**

Major online databases *Scopus*, *Psychlit* and *Pubmed* were searched for the terms “Community of Practice” (CoP) AND (Online OR Virtual OR Electronic) AND (health OR healthcare OR medicine OR “Allied Health”). Only peer-reviewed journal articles in English were selected. A total of 76 articles were identified, with 23 meeting the inclusion criteria. There were no studies on CoP or VCoP in General Practice training. The review was structured using a framework of six themes for establishing communities of practice, derived from a key study from the business literature. This framework has been used to analyse the literature to determine whether similar themes are present in the health literature and to identify evidence in support of virtual communities of practice for General Practice training.

**Results:**

The framework developed by Probst is mirrored in the health literature, albeit with some variations. In particular the roles of facilitator or moderator and leader whilst overlapping, are different. VCoPs are usually collaborations between stakeholders rather than single company VCoPs. Specific goals are important, but in specialised health fields sometimes less important than in business. Boundary spanning can involve the interactions of different professional groups, as well as using external experts seen in business VCoPs. There was less use of measurement in health VCoPs. Environments must be supportive as well as risk free. Additional findings were that ease of use of technology is paramount and it is desirable for VCoPs to blend online and face-to-face involvement.

**Conclusions:**

The business themes of leadership, sponsorship, objectives and goals, boundary spanning, risk-free environment and measurements become, in the health literature, facilitation, champion and support, objectives and goals, a broad church, supportive environment, measurement benchmarking and feedback, and technology and community.

General Practice training is under pressure from isolation and virtual communities of practice may be a way of overcoming isolation. The health literature supports, with some variation, the business CoP framework developed by Probst. Further research is needed to clarify whether this framework is an effective method of health VCoP development and if these VCoPs overcome isolation and thus improve rural retention of General Practice registrars.

## Background

General Practice is the cornerstone of an effective health system [1]. The Royal Australian College of General Practitioners defines General Practice as providing “person centred, continuing, comprehensive and coordinated whole person health care to individuals and families in their communities” [2]. High quality training is imperative to support this indispensable workforce, but in countries with low population densities, there are some inherent problems of professional and personal isolation for trainees in rural and regional areas. In Australia, the General Practice Training program involves multiple small training sites across a wide geographic area, which can be isolating for trainees [3]. To meet the ongoing needs of General Practice training and workforce, innovative solutions to overcome isolation need to be considered.

The provision of General Practice training and services in Australia is under pressure [4]. One of the causes of problems during General Practice training is isolation [3]. In the general medical population, isolation can lead doctors to reduce hours and move away from rural practice [5]. However, General Practice registrars may be under even greater stress than the general population of doctors, due to their clinical and training demands [6]. Online communities offer a means to reduce isolation [7]. In particular, virtual communities of practice are a type of online learning community that have been shown to be highly effective in large companies, improving knowledge sharing and thus overcoming professional and structural isolation [8,9]. Given the promise of online communities, this literature review will critically review the current evidence relevant to virtual communities of practice in General Practice training, identify evidence-based principles that might guide their construction and suggest further avenues for research.

Isolation can be subdivided into professional, structural and social isolation, although all three are often experienced concurrently [3]. Social isolation is more marked amongst rural General Practice placements, as trainees are away from their usual support network of friends and family. Professional isolation is also more common in rural areas, as trainees can be concerned about limited supervision and clinical back-up. Structural isolation, however, is common across all training placements. Structural isolation can result from consulting alone in a consultation room, as opposed to the team environment of the hospital. Social isolation can be described as a form of loneliness [10]. However, professional isolation is linked to a lack of knowledge sharing activities such as networking, tacit knowledge sharing and mentoring [11]. The result of these barriers to knowledge sharing can be ‘terrifying’, when there are serious health decisions to be made, as the following trainee describes.

In an interview study of General Practice trainees conducted in Australia in 1999, one trainee said “I found it unbelievably stressful starting in General Practice … country GP [was] always what I wanted to do. Got there—and I was shocked to find that I found it terrifying, isolating, extremely isolating…Just to have gone from a setting where you were working with colleagues constantly … so GP work is a big change. Sitting in one room.” [3].

Isolation has implications for the health system, as well as being a negative experience for the trainee. In Australia in 2008, GP registrars comprised 11% of the rural and remote workforce. However retention of registrars in rural areas continues to be a problem, with only 27% of previous Rural Pathway registrars (trainees committed to extra rural training) still working in rural practice in 2008 [12]. These problems are not confined purely to rural registrars or to Australia. In the US, a survey of 1700 physicians illustrated that stress and mental health issues, of which isolation is a component, can lead to physicians considering reduction in work hours, change of job or reduction in patient contact [5]. Effective means of overcoming isolation are urgently required to meet the needs of trainees and the health system.

Increasingly, people are using social networking tools to overcome personal and professional isolation by building relationships. Facebook alone now has over 845 million active users^a^ while LinkedIn has 150 million^b^ . A study of US college students found that usage of Facebook correlated with increased ‘social capital’ [7]- a term that broadly describes social relations that have productive benefits [13]. Not only was there a strong association between Facebook use and the formation and maintenance of social networks at a time when young people are often moving away from home and into a new phase of their lives, the findings also suggest that the benefits may be highest amongst students with low self-esteem and low life satisfaction. This suggests that social networking might be beneficial to General Practice trainees, a similarly mobile group that must frequently relocate during training [3], and may be even more valuable to those most vulnerable to low self-esteem and low life satisfaction that can be associated with isolation.

This mobile group of General Practice trainees can be thought of as a ‘Community of Practice’. ‘Communities of practice’ are “groups of people who share a concern or a passion for something they do and learn how to do it better as they interact regularly” [14]. The theory underpinning the idea describes master-apprentice learning, in which novices work alongside experts, gradually developing their understanding with explicit and implicit guidance from others in the community according to the norms of the group. In this interaction, those with greater expertise also gain knowledge. This form of learning community also incrementally builds a stock of knowledge resources for the community over time [15,16]. Although the research underpinning the theory of communities of practice was conducted in Yucatan midwives, US naval quartermasters and apprentice butchers, its appeal has spread.

The widespread growth of the internet in the late 1990s led to considerable interest in combining online tools with communities of practice theory to create ‘virtual communities of practice’. The main driver for these virtual communities of practice has been to connect people not located in the same place at the same time, thereby creating networks of people with common interests who are geographically dispersed. Virtual communities of practice have been successfully adopted by business, with significant interest from the education sector as well [17,18].

In the education sector, there is a wealth of literature on online and virtual communities of practice but little systematic review evidence [18,19]. Single study evidence is plentiful. For example a recent outcome study of an Internet-Based Master in Educational Technology demonstrates the efficacy of an online community of practice mixed with face-to-face teaching. The iMET program in Illinois graduated 85% of their 243 student within 3 years, compared with rates of 30% for other online Masters and 60% for some face-to-face [20].

In business, there is significant outcome data on the effectiveness of online communities of practice. In a systematic review of 43 studies, many with a mix of face-to-face and online support, communities were shown to decrease cost and increase innovation by allowing workers to effectively collaborate and share knowledge [21].

In business, as in health, experts play a significant role in developing the knowledge and skills of novices. Large volumes of information must be managed, employees in large companies can be spread over multiple sites and professional isolation must be overcome to improve knowledge sharing. Companies such as HP, Xerox and Caterpillar have implemented virtual communities of practice in which employees share knowledge online, sometimes mixed with face-to-face interaction [17].

In the health sector, communities of practice also show promise, but systematic reviews so far are inconclusive [21]. Since the most recent review [21], however, there have been some positive examples of communities of practice. For example, a UK Stroke service was redeveloped using a face-to-face community of practice model to set up a stroke unit and implement best practice. As a result, the service moved from the bottom 5% to the top scoring service in four years [22]. This potential has been recognised by other researchers, for example by the Montreal Stroke Network, which is planning a series of trials around an e-collaborative platform using Communities of Practice theory for knowledge sharing on best practice in stroke care. Despite these positive indications, there are still significant questions about the potential for virtual communities of practice to help build a healthy and effective General Practice workforce by overcoming isolation in training.

This paper provides a critical review of current research literature to determine what, if any, evidence there is for virtual communities of practice in General Practice training. In addition, this review identifies evidence-based guidelines for developing virtual communities of practice from the wider research literature which could inform implementation in General Practice training.

## Methods

A comprehensive literature search of the databases *Scopus*, *Psychlit* and *Pubmed* was conducted using the terms “Community of Practice” (CoP) AND (Online OR Virtual OR Electronic) AND (health OR healthcare OR medicine OR “Allied Health”). Only peer-reviewed journal articles in English were selected. There was no date range limitation applied due to the need to identify all potentially relevant studies from a small body of literature. The further inclusion criteria required that journal articles include primary research and involve virtual communities of practice and human clinical healthcare. Exclusion criteria eliminated opinion pieces, conference papers and unpublished theses. Studies with patients as participants were excluded as this literature review focuses on professional education, not patient management. Articles involving the higher education teaching or research sectors were also excluded, as these are distinct from clinical healthcare. Each article was then read in full to confirm compliance with the inclusion criteria. References were searched to identify additional relevant studies.

The search returned 97 articles. Duplicates were removed, leaving 76 articles. References were searched, returning one extra article. Of the 77 articles, 22 articles met the inclusion criteria. The 55 articles excluded were conference papers/theses, ‘community’ or ‘community of practice’ but not ‘virtual community of practice’, articles from outside human clinical healthcare education, including university students, research, veterinary science and business, studies involving patients, opinion pieces, IT semantic articles, unrelated articles, and a study proposal with no data.

None of the 22 relevant articles were specific to General Practice training. Most articles had small sample sizes and a variety of methodologies, with a number of descriptive studies. Because of this limited empirical base, this literature review is descriptive, although a formal count of each theme’s appearance in each paper was also performed.

There is a wealth of business related literature on CoPs and VCoPs. The strength of the business literature is the concrete outcomes that have been demonstrated as a result of using the CoP theory within a business. These outcomes include lower costs, lower lead time to market and saving of labour hours/year. For this reason the authors looked at the recent business literature for a potential CoP or VCoP model that might be applicable to the health sector. In a recent literature review, Agarawal and Joshi [9] cite Probst and Borzillo’s model [8]. The model, presented in their article “ Communities of Practice- Why they succeed and why they fail” was noted by the authors of the current literature review to be well structured, well supported, simple and yet comprehensive. It summarised, in a useful way, the themes that the authors had noticed emerging from the health literature. Many of the CoPs were also VCoPs, although a subset analysis was not done. A final strength of the model was the large amount of empirical data, in reputable companies, on which it was based; 57 CoPs in companies including Oracle, Siemens and IBM were reviewed.

The Probst and Borzillo model has been used to analyse the literature to determine whether similar themes are present in the health literature and to identify evidence in support of virtual communities of practice for General Practice training.

## Results and Discussion

Probst and Borzillo propose ‘ten commandments’ for effective communities of practice and suggests five key reasons for failure [8]. The researchers identify six key themes important to the establishment and maintenance of successful communities of practice: Leadership, Sponsorship, Objectives, Boundary Spanning, Risk-free environment and Measurements. These themes are explained and expanded upon as each theme is explored in relation to the literature identified for this review.

It must be noted that most of these studies are qualitative and there is varied statistical analysis and methodology reporting (Table 1). These papers have been read extensively and, where comments or discussions or conclusions from data, or from the project being discussed, are made, then these items are matched against the themes in Probst and Borzillo’s framework (Table 2). This is not an assertion that these themes have been formally studied as outcomes for each study. The additional themes of ‘Technology” and “Community” barriers and enablers have been included to cover a number of similar themes in these studies.

**Table 1:** Study summary

**Author**	**Approach**	**Brief description**	**Data Collection**	**Participants**	**Statistical analysis**	**Themes generated by primary data**
Andrew 2009	Informal case study	Nursing academics online support site iCoP	Analysis of weblog posts	14 nursing academics	None.	L,O,S,B,M,T
Booth 2007	Action research- mixed methods	Constructing evidence-based nursing care guidance for gerontological nurses using CoP and Virtual College	Focus groups, telephone interviews, analysis of online archives and documentary outputs	58 (30 in first CoP, 28 in second CoP)	None reported.	L,O,S,B,M,T,C
Brooks 2006	Case study organizational research	Study of midwives as knowledge workers using online forum (subset of AEC project)	Interviews, focus groups and analysis of online forum postings	42 participants	Usage, message types- coded by 3 researchers. SPSS gave percentages.	L,S,O,R,M,T,C
Brooks 2006	Qualitative study	Assisted Electronic Communication (AEC) project for nurses, using an online forums	15 interviews and analysis of online forum postings	44 participants and 193 messages	Communications coded into categories. Percentages presented. Interview data presented	L,S,T,C
Curran and Murphy	Mixed methods	VCoP of Emergency clinicians in Canada	Online posting analysis and ‘post’ survey	270 ED clinicians	Percentages and descriptive statistics of content and surveys	L,B,M,T,C
Falkman 2008*	Mixed methods	SOMWeb, an online CoP for oral surgeons in Sweden	Interview, online message review, meeting observation and survey.	90 members 24 survey responses, 9 interviews and 10 meetings observed.	Interviews with quotations.	L,O,B,T,C
Falkman 2008**	Mixed methods	Another paper on SOMWeb – an online CoP for oral surgeons	Online questionnaire and interviews	Not reported	Not reported	L,O,T
Hara 2007	Mixed method case study	Listserv for nurses in USA	Analysis of online postings and interviews	27 interviews	Qualitative review of observations and interviews, descriptive statistics for types of activity and knowledge data.	L,O,R,M,T,C
Ho 2010	Project description	Electronic detailing project on diabetes (TEAD)	Description of electronic detailing project, mentions surveys and data collection.	Not reported. No formal data presented	None presented	L,O,B,T,C
Li 2009	Systematic review	Review of effectiveness of business and healthcare CoPs	Electronic database search	18 primary business studies, 13 primary healthcare studies. Qualitative studies. No assessment of quality of studies	Published as a systematic review of qualitative data. No theme counts or statistical analysis	L,O,C
Nagy 2006	Case report	An online PACS (radiology system administrator) community	Description of successful project	Site statistics- 2500 members. No formal data.	None.	L,O,R,T,C
Penn 2005	Project description	An online suicide prevention site for mental health workers	Description of design and background and some initial findings of ACROSSNet	No data- project description only.	None.	L,O,B,R,T,C
Perotta 2006	Qualitative	An online psychology community in Italy	Analysis of online postings	20 discussion topics with average 12.5 postings.	Theme count and interviewee quotations	O,B,C
Poissant 2010	Research protocol	The development of an e-collaborative platform for the Montreal Stroke Network	Not applicable	Not applicable	Not applicable	L,O,B,S,T,C
Poole 2008	Action research	Women’s Health VCoPs in British Colombia	Outcomes of webinars and description of resulting presentations and materials	Six VCoPs. Total participants not reported.	No formal analysis of outcomes	L,O,S,B,T,C
Rolls 2008	Quantitative	Intensive Care Unit clinician network in Australia	Survey study	Online survey. 113 respondents (26% response rate)	Response percentages, total numbers and comment on statistical significance but method not reported	L,O,S,B,T
Russell 2004	Qualitative	CHAIN an email based evidence service in the NHS, UK	Posting analysis, feedback both active and unsolicited, interviews	2800 members, 102 messages and 22 requests in study period. Three focus groups x 15 members each.	None. Feedback examples given.	L,O,S,B,T,C
Sharma 2006	Qualitative	Study of an online incident reporting system for anaesthetists in UK	Interviews	10 respondents, three interviews each	Discussion of interview outcomes. No quotations. No method of interview analysis reported	L,S,R,T,C
Thomas 2010	Case study	GAPS project on sharing family planning information for WHO	Moderated discussions analysed as part of case study	273 members of network. Three moderated forums analyzed. Participant numbers not reported.	Themes from discussions reported. No quotations or theme counts. Methodology of theme generation not reported	L,O,S,B,C
Tolson 2005	Qualitative	Nurses used an online forum (Virtual College) for gerontological nursing	Interview study	15 nurses, 20–30 minutes each interview	Qualitative analysis with methods reported-cognitive mapping performed to generate themes. Five themes generated.	L,O,S,B,R,T,C
Tolson 2008	Mixed methods	Review of effect of a Virtual College and CoP on implementation of Best Practice Statements	Focus groups, pre and post intervention audits	24 nurses. 476 ‘pre’ audits, 344 ‘post’ audits. Focus groups- numbers not reported.	Statistical analysis of audits using t tests. Focus group quotations.	L,O,S,B,R,M,T,C
Valaitis 2011	Q methodology	Explored views of nurses using online CoP to support practice in homeless populations.	Online survey and focus groups	66 statements collected from survey and groups, refined to 44. 16 nurses completed the Q-sort activity	By-person factor analysis of Q-sort.	L,E,T,B

*Key: L = Leadership, O = Objectives, S = Sponsorship, B = Boundary Spanning, R = Risk-free environment, M = Measurements, T = Technology, C = Community*.

Brooks 2006* = Nursing and Health Management and Policy.

Brooks 2006** = International Journal of Nursing Studies.

Falkman 2008* = Journal of Medical Internet Research.

Falkman 2008** = Studies in Health Technology and Informatics.

**Table 2:** Theme count

**Probst and Borzillo Theme**	**Theme description**	**Comments supportive of theme**	**Comments non-supportive of theme**	**Supportive count**	**Negative count**	**Total count**
Leadership	The organisation can designate leadership roles to motivate community members to collaborate	Andrew 2009, Booth 2007, Tolson 2005, Tolson 2008, Brooks 2006**,Brooks 2006*, Curran 2009, Falkman 2008**, Falkman 2008*, Hara 2007, Ho 2010, Li 2009, Nagy 2006, Penn 2008, Russell 2004, Poissant 2010, Poole 2008, Thomas 2010	Booth 2007, Sharma 2006, Valaitis 2011, Rolls 2007	18	4	22
Objectives	Clear objectives provide members with responsibilities and motivates them to contribute more actively	Andrew 2009, Booth 2007, Falkman 2008**, Falkman 2008*, Hara 2007, Ho 2010, Li 2009, Penn 2005, Russell 2004, Poissant 2010, Poole 2008, Thomas 2010, Rolls 2007, Perotta 2006, Tolson 2005, Tolson 2008	Brooks 2006*, Nagy 2006 Penn 2005	15	3	18
Sponsorship	Senior executives need to provide sponsorship to help communities reach their full potential	Andrew 2009, Booth 2007, Tolson 2008, Brooks 2006**,Brooks 2006*, Russell 2004, Poissant 2010, Poole 2008, Sharma 2006, Thomas 2010, Tolson 2005, Rolls 2007		12	0	12
Boundary Spanning	Boundary spanning enables members to engage in internal and external benchmarking practices	Andrew 2009, Booth 2007,Falkman 2008*, Tolson 2008, Tolson 2005, Curran 2009, Ho 2010, Penn 2008, Russell 2004, Poole 2008, Poissant 2010, Rolls 2007,Thomas 2010	Andrew 2009, Perrotta 2006, Valaitis 2011	12	3	14
Risk-free environment	COPs should be used as an especially valuable opportunity to express and test ideas in an informal and risk-free environment, thus requiring a strong degree of safety and intimacy between members	Tolson 2005,Tolson 2008, Brooks 2006*, Hara 2007, Nagy 2006, Penn 2008, Sharma 2006	Penn 2008, Valaitis 2011	6	2	8
Measurements	Empirical evidence suggests the use of measurements to assess the value of communities of practice	Andrew 2009, Booth 2007, Tolson 2008, Brooks 2006*, Curran 2009, Hara 2007		6	0	6
Technology ***	Technology enablers (points supportive of this theme) and barriers (points against this theme)	Andrew 2009, Falkman 2008**, Falkman 2008*, Booth 2007, Tolson 2005,Tolson 2008, Brooks 2006**, Brooks 2006 *, Hara 2007, Ho 2010, Nagy 2006, Penn 2008, Russell 2004, Poole 2008, Sharma 2006, Valaitis 2011, Rolls 2007, Poissant 2010,	Andrew 2009, Brooks 2006**, Brooks 2006*, Curran 2009, Sharma 2006, Tolson 2005, Valaitis 2011	16	7	23
Community ***	Points which build community (supportive) and reduce community (against)	Booth 2007, Poissant 2010, Thomas 2010, Falkman 2008*, Brooks 2006**, Brooks 2006*, Poissant 2010, Rolls 2007, Curran 2009, Hara 2007, Ho 2010, Li 2009, Nagy 2006, Penn 2008, Russell 2004, Thomas 2010, Perotta 2006, Poole 2008, Tolson 2005, Tolson 2008	Hara 2007, Sharma 2006	19	2	21

Brooks 2006* = Nursing and Health Management and Policy.

Brooks 2006** = International Journal of Nursing Studies.

Falkman 2008* = Journal of Medical Internet Research.

Falkman 2008** = Studies in Health Technology and Informatics.

*** = Technology and Community are two extra themes added by the authors of this literature review and do not appear in Probst and Borzillo’s model (See Table 3).

### Theme 1: Leadership

#### Probst: The organisation can designate leadership roles to motivate community members to collaborate

Almost every study in this review commented on leadership, facilitation or moderation [21,23-38]. Previous studies have commented on the lack of clarity around these terms in virtual communities of practice [21]. In this review, it appears that these roles, whilst overlapping, are different.

### Facilitators/Moderators

The most common role described in the studies was of the facilitator or moderator. This role may arise in several ways. The originator of the group may end up being the initial leader and facilitator [23]. The facilitator may be appointed by the originators of the group [24-26] or the facilitators of the group may arise spontaneously [24].

If they arise spontaneously, then these moderators or facilitators tend to be part of the ‘core group’ which also characterises these virtual communities [23]. The ‘core group’ consists of a minority of active users, whilst often the majority is passive [25,26]. Despite this passivity, these users are still seen as benefiting from the network as ‘legitimate peripheral participants’. As one GP put it, I have not used CHAIN much but it is a security blanket!” [26].

The tasks of the facilitator and moderator are, as Probst described, to improve collaboration [27,28], but can also include making sure the rules of engagement are clear, keeping discussions focussed and processing memberships [23,26,27,29].

There is some controversy about ongoing facilitation. One researcher believed that these networks can be self-sustaining [23], one found that it was definitely not [30], however most simply used facilitators, or had facilitators emerge, throughout the projects.

### Leadership

In one study without formal facilitators, ‘leaders’ emerged. This ‘emergence’ demonstrated the opportunity for horizontal leadership to occur in VCoPs, in which marginalised or junior members of staff have the chance to emerge into leadership roles, potentially taking forward actions that arise from discussions [27].

In the same online midwifery forum, more senior nurses used their postings to praise other contributors and to validate the use of the forum, successfully encouraging usage. However, praise online actually fits better with the role of a moderator and from the perspective of Probst’s thematic analysis, the ‘leadership’ shown in validating the use of the forum by the organisation may fit better under ‘sponsorship’ [24].

Probst tells us that the role of the leader is in promoting collaboration [8]. However the definition of leadership in the articles reviewed is controversial. Li’s systematic review highlights the fact that the role of leader and facilitator may be separated or performed by the same person [21]. In terms of roles, in the articles reviewed it appears that it is actually the facilitator and moderator who promote collaboration. Leadership, when implying validation by the organisation, can actually be seen as equivalent to Probst’s ‘Sponsorship’ or the display of executive approval for the network. The main importance of the leader found in this review is in the initiation of the community. In many of these studies that role was actually performed by the study organisers [30,31,39]. In studies in which the study organisers are not the leaders, then this concept of leadership and initiation merge with Probst’s concept of sponsorship.

### Theme 2: Sponsorship

#### Senior executives need to provide sponsorship to help communities reach their full potential

In business, Probst’s finding was that effective CoPs had a sponsor, or senior executive, who sanctioned the CoP. There was then a leader that drove the community [8].

The findings in the current literature review were that, in fact, in health the agenda is usually driven by the organisation attempting to start the community and/or the researchers founding the community. It is then the moderators and active group that continue to stimulate and promote knowledge sharing.

Sponsorship, initiation, vision or leadership was evidenced in many of the studies, as the groups were collaborations between stakeholders that were forming a network to solve a problem. Ultimately, someone had to start the network, then continue to support its activities. For example, the CHAIN network of evidence in the UK is part of the NHS Research and Evaluation network, ICUConnect is part of the ICU Monitoring Unit and the proposed e-collaborative platform for the Montreal Stroke Network is formed from a number of state and national stakeholders [26,29,32].

Once created, ongoing organisational support was essential to the success of projects. This was demonstrated well in a group of gerontological nurses that needed ongoing support from high-level nurses to legitimise work-based learning, before the use of the online environment was accepted [39].

Whilst sponsorship describes the process of the corporate world well, in the health context there are some differences. Mostly, the networks have an initial purpose of knowledge sharing that supports the organisation, or the researchers’ study, and thus are a collaboration of multiple stakeholders such as a health service, the researchers and clinicians, rather than the domain of a single company.

### Theme 3: Objectives

#### Clear objectives provide members with responsibilities and motivate them to contribute more actively

Each VCoP studied had an objective, however these objectives ranged from clear and specific to broad. The success of networks with specific objectives initially appears to support this statement [24,25,31,34,39]. For example, the development of evidence-based ‘best practice’ statements for gerontological nurses in Scotland led to the better uptake of evidence-based practice, using a Virtual College and CoP. However, a number of networks had broad objectives within a specialised group of practitioners and were also successful [23,24,34]. For example, Nagy’s network for PACS online radiology systems had a broad objective to “facilitate and accelerate PACS through education and communication”. Within that framework, users developed their own goals and content through posted queries and responses. A similar pattern was found in Brooks’ midwifery forum [27].

However, when a busy psychologists’ network was reviewed for the outcome of ‘professional identity creation’, there was less success. The network had not been set up for this, and perhaps its broad goal of providing a ‘meeting place where ..professionals…can establish valuable relations; sharing experiences information and practices...’ contributed to the lack of specific identity formation [35]. Also, a network of nursing academics experienced some problems with lack of focus [30].

Probst describes clear objectives and sub-objectives for CoPs. For example, a car manufacturer may have a broad objective of improving engine performance, with sub-objectives around building and exchanging technical knowledge around each of the engine parts (valves or internal combustion for example). The findings from this review are that specific objectives are helpful although, particularly in a specialised area such as midwifery or radiology systems, some networks succeed without a high degree of clarity around their goals.

### Theme 4: Boundary spanning

#### Boundary spanning enables members to engage in internal and external benchmarking practices

Most groups in this review benefited from a heterogeneous make-up, although there were some problems. In almost every study, there were either a variety of practitioner types, or a variety of organisations participating. Booth found that linking CoPs in different sites via the virtual college accelerated their guideline development process for nurses [31] and Curran’s rural emergency departments benefited from their city cousins sharing expert knowledge and from the use of knowledge experts [40]. The evidence-based CHAIN network in the UK described the effective knowledge sharing between groups as a demonstration of strong and weak tie theory [26]. In this instance, strong ties are between users that know each other best, but weak ties between users only distantly acquainted or introduced via the network led to the greatest knowledge sharing.

However, if the group is too heterogeneous, there can be problems, as there is either not enough overlap for effective communication or antagonistic viewpoints between competing groups [30,35].

Probst describes members of CoPs either being fed with external expertise, or making use of other CoPs either within, or from without, the CoPs company. This view differs from the health experience in that often these networks do not originate within a single ‘company’ or stakeholder. The boundary spanning occurs through the interaction between either different professional groups or different organisations, or both, whilst some used external experts.

### Theme 5: Risk-free environment

#### COPs should be used as an especially valuable opportunity to express and test ideas in an informal and risk-free environment, thus requiring a strong degree of safety and intimacy between members

A risk-free environment came through as important in this review. Moderators were encouraged to enforce rules of no offensive language and ‘model citizen behaviour’ [23,27] and protocols were developed about how users are to behave online with expectations of themselves and each other [34].

In addition to lack of risk, positive reinforcement was also important, along with a non-hierarchical atmosphere. One nurse said “I think if you keep encouraging people they will think and be creative” [39], whilst another commented that “It’s (the online environment), you know, a free atmosphere; to be able to do it without any comeback” [36].

A demonstration of the risks that users fear was the fact that Penn’s Suicide Prevention network had still not progressed to its original goal of online psychiatry advice due to legal concerns [34]. In addition, in an online anaesthetic network reporting on critical incidents, it was felt that some of the lack of reporting was due to the general culture of low reporting of incidents. This network also commented that users requested anonymity as an option, likely for the same reason [41]. Probst’s review demonstrates that a risk free environment is important in business to encourage growth. In health, although an environment must be risk free, it should also be positive and encouraging. This type of environment builds trust and thus improved communication.

### Theme 6: Measurements

#### Empirical evidence suggests the use of measurements to assess the value of communities of practice

There was very little formal measurement identified in this review. One study found that regular feedback provided to participants assisted them in decision-making [31]. However, several studies commented on the value of informal ‘benchmarking’ or ‘validation’ of their own practice against that of other users and organisations [27,39,40], while other participants generated their own ‘closing the loop’ of actions resulting from the online discussions [24].

#### Measurement, benchmarking and feedback

The VCoPs in Probst’s review had more measurable goals, such as cost reduction or product improvement. However, he still notes that members posting online ‘stories’ of how their experiences have led to positive change motivates other members. In the health context, these measurements may be more likely to be member-generated, including benchmarking of practice or having feedback about organisational changes that have been triggered as a result of the discussion, rather than formal manufacturing targets.

### Technology and community features

Whilst not specifically addressed by Probst and Borzillo, a number of other themes were found in this literature review, which have been grouped under the headings Technology and Community Features.

### Technology

Making the technology easy was commonly cited as highly important. The concept of ‘easy’ included ease of use, ease of access and flexibility of options for communication [24,27,28,30,34,37,41].

Communication options in most studies included an asynchronous method, either by email or discussion boards [23,24,26,28,34,37,39,42], while some studies used these with a mix of features including chat, content sharing and synchronous web-meetings [23,34,35,39]. Email reminders were also suggested to be useful [26,37,41].

Whilst the previous features were more uniform, a number of areas were controversial. Some studies used passwords [28,42] though lost passwords and online delivery created barriers for others [37,39,40]. The online environment was of real benefit to most [24,27,35], though one study found that the culture of face-to-face interaction amongst nurses was a barrier to use of online environments [30]. Lastly, training was mentioned as necessary by some [39] whilst others aimed to avoid training through simplicity of design [24].

Ease of use is paramount in any online community. Communities should offer asynchronous communication methods such as email and discussion boards and may consider other options such as chat and content repositories. When setting up a community, consideration needs to be given to the pros and cons of passwords, access, identification and training.

### Community features

Effective communities of practice result in knowledge sharing [15]. This knowledge sharing can be encouraged by voluntary involvement, as self-selection appears to encourage users that are willing to share knowledge to participate [27,28]. A particular feature of the CHAIN network of evidence in the UK is the reciprocity of members, that is the generosity of members when responding to queries from others [26]. However, whilst this active membership is essential, passive users can still be seen as Lave and Wenger’s ‘legitimate peripheral participants’, gaining support from watching the ‘expert’ users [25,26]. The validation of each others’ practice and a desire to understand current knowledge are other factors that help sustain an online CoP [24,27,40].

Whilst online membership is helpful in overcoming barriers of geography and time [24,27,30], bonds can be strengthened through face-to-face meetings [31,32]. In fact, one network started online, with physical chapters developing as a result [23].

Communities can help professionals overcome isolation through connecting with colleagues and sharing knowledge [27,38]. One nurse said “I feel fairly isolated [because] I don’t have many peers (advanced practice nurses) in my organisation. The listserv helps give me ideas when I have no-one else to bounce ideas with in my hospital”.

In addition to the features mentioned by Probst and Borzillo, self selection, a desire to knowledge share and the blending of face-to-face and online involvement are desirable. It is worth noting that it is not just the active users that benefit from membership in such communities.

### Implications

From this review it can be seen that there may be a role for VCoPs in general practice training, although a planned approach to research is needed. A VCoP for general practice training may decrease the social, structural and professional isolation aspects of training, thus improving trainees’ sense of connectedness and improve their knowledge sharing opportunities. The benefits of these outcomes could include higher general practitioner trainee satisfaction and knowledge, particularly whilst in rural placements, with implications for possibly helping to overcome workforce shortages and quality health care delivery in these areas.

Another potential benefit of a VCoPs for general practice training is that VCoPs can offer the potential to make invisible work visible. This might enable areas of practice that have traditionally occupied lower status in general practice to gain significance as members communicate their experiences. An example of a VCoP for general practice trainees could include online expert medical moderators facilitating case discussions, answering questions and helping to build a shared knowledge resource for trainees. During this process, under-represented or marginalised areas such as workers’ compensation related illness or youth mental health may be highlighted in discussion, thus raising their profile as well as providing practical tips for trainees with little exposure to these difficult areas.

### Limitations

There are a number of limitations to this study. Firstly, the initial model is drawn from the business literature, with business outcomes in mind. In health, CoPs often involve several organisations, rather than one business. They may also be non-profit and the outcomes being measured may be more related to clinical care delivery or knowledge sharing and overcoming professional isolation. It was also unclear in the Probst and Borzillo model how many of the CoPs were in fact VCoPs and there was no subset analysis on this differentiator, which is noted in the Probst and Borzillo paper.

Secondly, the overall data quality of many of these papers is limited and in particular there is very little rigorous outcome data. Future studies must include an examination of efficacy in addition to qualitative review.

Finally, the themes that have been generated from each paper are not formal themes that have been evaluated in each paper. In many cases they are drawn from descriptions of the project or interpretations of the data by authors, but with variable data quality (see Table 2).

## Conclusions

Good General Practice is core to good care delivery and needs to be maintained by a high quality training of new general practitioners. However, General Practice registrars face a number of pressures, including professional, structural and geographical isolation.

Virtual communities of practice in business have been shown to improve knowledge sharing and overcome geographical boundaries, essentially overcoming professional and structural isolation. There are some promising signs in the health literature that VCoPs may help to overcome isolation, but studies are few and there is no systematic review evidence.

This review shows that a highly cited framework for VCoP development in the business literature could be applied to the current health literature, with some amendments (see Table 3). As a result, further research is needed to validate whether this framework is an effective method of health VCoP development, whether such a VCoP is effective in overcoming isolation in General Practice training and, if so, whether VCoPs could be a tool for improving General Practice training and retention, particularly in rural areas.

**Table 3:** Proposed health VCoP framework

**Probst’s business CoP framework**	**Proposed health VCoP framework**
**Leadership**	**Facilitation**
The organisation can designate leadership roles to motivate community members to collaborate	Facilitators promote engagement and maintain community standards
**Sponsorship**	**Champion and Support**
Senior executives need to provide sponsorship to help communities reach their full potential	The network needs to have an initial stakeholder champion, with stakeholder support
**Objectives and Goals**	**Objectives and Goals**
Clear objectives provide members with responsibilities and motivates them to contribute more actively	Clear objectives provide members with responsibilities and motivates them to contribute more actively
**Boundary Spanning**	**A Broad Church**
Boundary spanning enables members to engage in internal and external benchmarking practices	Consider involving different, overlapping but not competing, professional groups, different organisations and external experts. However make sure the church is not too broad....
**Risk-free environment**	**Supportive environment**
COPs should be used as an especially valuable opportunity to express and test ideas in an informal and risk-free environment, thus requiring a strong degree of safety and ntimacy between members	Health VCOPs should promote a supportive and positive culture that is both safe for members, and encouraging of participation
**Measurements**	**Measurement, Benchmarking and Feedback**
Empirical evidence suggests the use of measurements to assess the value of communities of practice	Health VCoPs should consider measurement as a factor in their design, including benchmarking and feedback
	**Technology and Community**
	Online CoPs should ensure ease of use and access, along with asynchronous communication. Other options including chat and meetings can also be considered, along with the need for training.
	Communities are more likely to share knowledge when there is a mixture of online and face-to-face meetings, members self select, and both passive and active users are encouraged.

## Endnotes

^a^Facebook Fact Sheet, website press release [ http://newsroom.fb.com/content/default.aspx?NewsAreaId=22]

^b^LinkedIn press release [ http://press.linkedin.com/about

## Abbreviations

CoP: Community of Practice; VCoP: Virtual Community of Practice.

## Competing interests

Dr Stephen Barnett is the Medical Director and part-owner of www.e-healthspace.com.au an online community for Australian doctors.

## Authors’ contribution

SB- conceived the study and did the majority of data analysis and writing of the paper. SJ- assisted with conception and design of the study and provided ongoing review of drafts. DI- provided assistance with design of the study and review of drafts. SuB- assisted with design particularly around communities of practice and assisted with drafting the manuscript. AB- assisted with conception of the study, and review of drafts. All authors read and approved the final draft.

## Authors’ information

Dr Stephen Barnett is a Senior Lecturer in General Practice and Educational Technology at the Graduate School of Medicine, University of Wollongong, New South Wales, Australia. He has an interest in the intersection of technology and health care education and delivery. He is active as a General Practitioner and General Practice registrar supervisor.

## Pre-publication history

The pre-publication history for this paper can be accessed here:

http://www.biomedcentral.com/1471-2296/13/87/prepub
